# High-Throughput Heterogeneous Integration of Diverse Nanomaterials on a Single Chip for Sensing Applications

**DOI:** 10.1371/journal.pone.0111377

**Published:** 2014-10-28

**Authors:** Samuel MacNaughton, Srikanth Ammu, Sanjeev K. Manohar, Sameer Sonkusale

**Affiliations:** 1 Nanolab, Department of Electrical and Computer Engineering, Tufts University, Medford, MA, United States of America; 2 Department of Chemical Engineering, University of Massachusetts Lowell, Lowell, Massachusetts, United States of America; Texas A&M University, United States of America

## Abstract

There is a large variety of nanomaterials each with unique electronic, optical and sensing properties. However, there is currently no paradigm for integration of different nanomaterials on a single chip in a low-cost high-throughput manner. We present a high throughput integration approach based on spatially controlled dielectrophoresis executed sequentially for each nanomaterial type to realize a scalable array of individually addressable assemblies of graphene, carbon nanotubes, metal oxide nanowires and conductive polymers on a single chip. This is a first time where such a diversity of nanomaterials has been assembled on the same layer in a single chip. The resolution of assembly can range from mesoscale to microscale and is limited only by the size and spacing of the underlying electrodes on chip used for assembly. While many applications are possible, the utility of such an array is demonstrated with an example application of a chemical sensor array for detection of volatile organic compounds below parts-per-million sensitivity.

## Introduction

Nanomaterials have been the subject of much attention in recent decades for their promise in realization of high performance electronic and optical devices, or ultrasensitive biological, chemical and physical sensors. Nanomaterials demonstrate extreme dimensionalities, meaning that the materials have extremely high surface-to-volume ratios. This affords a higher sensitivity compared to bulk materials as interactions at the surface of the material (*e.g.* with gas molecules) can affect the material’s properties as a whole. However, despite their sensitivity, the vastly differing synthesis methods of nanomaterials presents a difficulty in creating large arrays of differently synthesized nanomaterials on a single substrate. In this work, the three most prevalent families of chemiresistive nanomaterials, both organic and inorganic, are integrated onto a single chip. The nanomaterials chosen for this work come in different shapes, such as nano-platelets of reduced graphene oxide (rGO), nanotubes of carbon (CNT), nanowires of copper oxide (CuO) and nanostructured polypyrrole (PPy). This is the first time that a heterogeneous integration of such diverse nanomaterials has been achieved in a single chip-scale platform. While many applications are possible that can utilize this paradigm of high throughput assembly of different nanomaterials on a single chip, we demonstrate its utility for realization of a chemical sensor array. Such a sensor array chip would be of great interest in the areas of food service, medicine, environmental monitoring, and military/security.

We briefly present an overview of the four different nanomaterials utilized in this paper for realization of this chemical sensor array. Graphene nano-platelets and carbon nanotubes represent two of the four sensing nanomaterials used. Graphene is a promising two-dimensional allotrope of carbon in which the carbon atoms are arranged in a hexagonal lattice with potential applications in energy storage, catalysis, electronic devices, as well as sensing [1], [2], [3]. Chemically-synthesized reduced graphene oxide (rGO) does not exhibit a chemically inert surface, but rather one with many dangling oxygen atoms and other functional sites such as alcohol and carboxyl groups. The sensing action is most often attributed to the gas phase analyte acting as an electron donor or receiver at the impurity sites, which then modulates the carrier concentration, and, thereby, conductivity of the material [4], [5], [6]. The 1-dimensional analog of graphene, carbon nanotubes (CNTs) have also long been investigated, for role of defects and electron exchange with and without functionalization, and reported sensitivities in the PPB range have been demonstrated [7], [8]. Metal oxides are the most common form of chemiresistive sensor: Tin oxide sensors (the Taguchi sensor) have long been used in a wide range of applications [9]. In this work, copper oxide nanowires are explored for their chemiresistive properties at room temperature. Copper oxide is an innately p-type semiconductor which has been the subject of limited studies for its gas sensing properties [10], [11]. Conductive polymers, including polypyrrole (PPy), have long been used as chemiresistors, and, more recently, nanostructured conductive polymers have been reported [12], [13], [14]. Conductive polymers are naturally non-specific and respond to a wide variety of gases, making them very popular elements in cross-reactive sensor arrays. Heterogeneous integration of this diverse family of nanomaterials in a single chip-scale platform can provide the necessary diversity and selectivity needed for cross-reactive chemical sensing of many different target analytes. In the past, this has been achieved by functionalizing and/or modifying elements within the same family of chemiresistors (i.e. carbon [15], [16], metal oxides [17], [18], [19], or conductive polymers [20], [21]). Recently, we showcased a sensing platform that had different types of metal oxide nanowires for gas sensing [22].

Scaling up to a larger array presents many practical challenges of their own: First, because the number of sensors is large, the fabrication of such an array necessitates a reproducible mechanism of high throughput and robust integration. Secondly, the simultaneous operation of many sensors in the array involves overcoming many difficulties in addressing and readout. For heterogeneous integration of different nanomaterials, contact printing or stamping have been proposed. [23], [24], [25] However, this strategy suffers from registration issues: successive layers can only be aligned to an accuracy of 2 µm. The minimum feature size exceeds many microns due to the mechanical considerations of the elastomeric stamp. These layer-by-layer strategies also do not allow cohabitation of diverse nanomaterials on the same layer; different nanomaterials can only be stacked and covered by successive passivation or spacing layers. Ideally, a strategy for the integration of heterogeneous materials into a massive array should allow for exceptional spatial precision, reproducible characteristics and parallel assembly/high throughput, while being applicable to a wide variety of nanomaterials. In this paper, we use a directed electric field assembly approach that allows for spatially localized, user controllable, directed assembly based on dielectrophoresis (DEP). DEP is the motion of a polarizable particle in a non-uniform electric field. The force experienced by the particle of arbitrary geometry can be expressed approximately by the following set of equations [26], [27]:
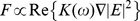
(1)

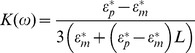
(2)

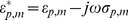
(3)where *ω* is the angular frequency of the applied field, *E* is the strength of the applied field, *L* is a geometry-dependent polarizability factor, ε_m_* and ε_p_* are the complex permittivity of the medium and particle respectively which is a function of *ε,* the permittivity, and σ, the conductivity.

**Figure 1:**
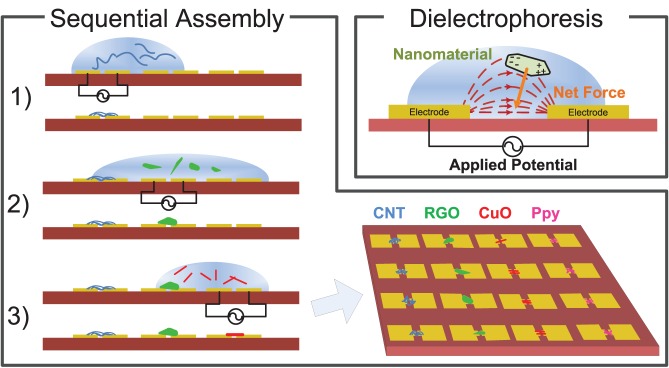
Sequential spatially controlled dielectrophoresis approach: By routing the assembly signal to many electrodes in parallel, many electrodes can be populated simultaneously. This allows the creation of massive, heterogeneous arrays. The premise of dielectrophoresis is shown in the upper right: a polarizable particle in a dielectric medium will experience a net force along the gradient of an applied field.

To use DEP for heterogeneous assembly, one can use a patterned array of electrodes driven by an alternating voltage source to generate arbitrary, user-defined non-uniform electric field for assembly to happen at specific locations. Figure 1 shows the proposed sequential spatially controlled dielectrophoresis approach for integration of four different nanomaterials. In this scheme, an AC signal is applied to the desired electrodes in the array, and the chip is immersed in a dispersion of a given nanomaterial. The entire process takes a matter of minutes. This process can be repeated with different dispersions successively to assemble many different nanomaterials in the same plane (or layer) in a high throughput manner which is not possible with micro (or nano) contact printing or other approaches. The fabrication of underlying chip with electrode pattern is the only part that is performed in cleanroom environment using top-down approaches for photolithography, while the rest of DEP assembly process is performed at room temperature in a modest cleanroom laboratory environment.

The presented method of assembly offers many distinct advantages compared with other strategies for assembly such as contact printing, inkjet printing or drop casting [28], [29], [30], [31], [32], [33]. First, every nanomaterial can be synthesized under their own optimal conditions without the need to be compatible with the fabrication conditions of another nanomaterial, and also separate from the conditions of the directed assembly process. Second, there are no special environmental controls; the process can take place on a bench top without any specialized equipment. It just needs a function generator for sourcing AC voltage and the dispersions of different nanomaterials to be assembled. The process takes minutes for each nanomaterial, and avoids time consuming preparation steps needed in other approaches. Lastly, the deposition of material is localized to a spatial location on the substrate defined by the electrode locations where the electric field is applied. With DEP, the electrodes and assemblies are naturally aligned, unlike other technologies where registration may be an issue. Previously, we had presented some preliminary work on the raw capability of dielectrophoresis for assembly and monitoring of carbon chemiresistors and copper oxide nanowires [34], and on the hybrid CMOS-nano integration of carbon nanotubes on a CMOS chip for gas sensing [35]. The resolution of the assembly defined as the spacing between different assemblies of nanomaterials, and the size of each assembly is dictated by the spacing and size of the underlying electrodes on chip. Prior work with DEP for assembly has shown that resolution ranging from meso- to nano- scale is possible using optical or electron-beam lithographically patterned electrodes on chip.

## Methods

**Figure 2:**
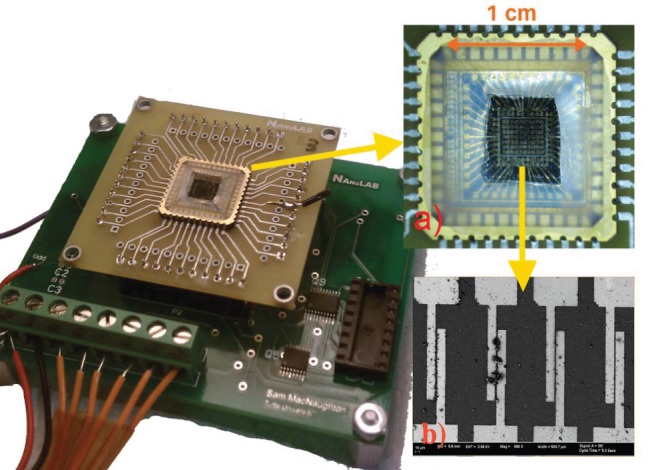
Printed circuit board for selectively addressing each electrode pair for DEP assembly and resistance readout. Inset (a) shows a magnified image of the semi-encapsulated packaged chip. Inset (b) shows a microscope image of a typical site for DEP assembly.

**Figure 3:**
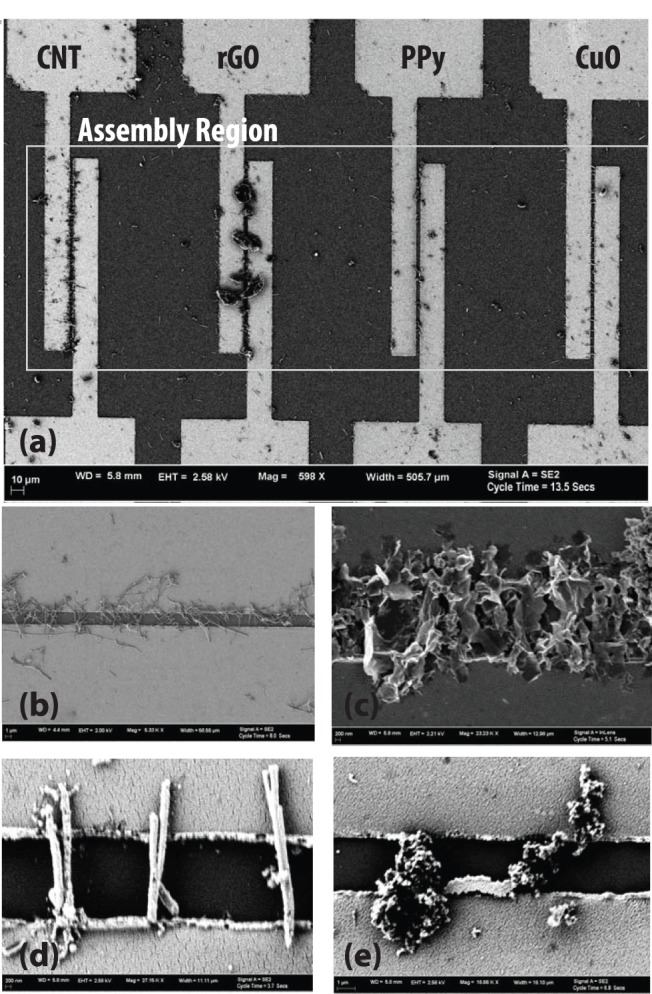
SEM images. (a) Showing four neighboring electrodes from an array assembled with CNT, rGO, PPy and CuO nanomaterials at different electrodes from left to right (b) close up of CNT assemblies (c) close up of rGO flake assemblies (d) close up of CuO nanowire assemblies and (e) close up of polypyrrole assemblies.

The platform for DEP assembly was created by standard photolithographic process on a silicon wafer with an insulating oxide surface. A single gold layer pattern contains all the electrodes and necessary trace routing for addressing each of 40 electrode pairs. There is a common ground electrode in every electrode pair, while the other electrode is routed to its own bond pad for addressing. An electrode with a 3 µm gap between electrodes (shown in Figures 2 & 3) was chosen as these dimensions gave the most consistent assemblies for different nanomaterials.

Individual 1 cm^2^ chips fabricated in this manner are then mounted into packages and wire bonded to establish electrical connections to each of the 40 microelectrode assembly sites distributed on the chip. The number of electrodes is limited by interconnect bottlenecks and the amount of pads in the lead frame. The chip is partially encapsulated in epoxy such that wire bonds are protected; yet the electrodes are still exposed for dielectrophoretic assembly and subsequent gas measurements (see Figure 2). This is accomplished by placing a PDMS block over the active area, using it to mask and protect this area while UV-curable epoxy is poured around it. Without this encapsulation, the wire bonds would not survive the immersion and drying cycles of DEP assembly. The final product is a chip that serves as a scalable platform for the creation of a heterogeneous array.

The processes used to create each nanomaterial used in the array are as follows: Reduced graphene oxide is produced in a process evolved from Hummer’s method [36]. In brief and in general, the process involves oxidizing graphite (thermally or chemically) and then chemically reducing the resultant graphene oxide to make graphene. The synthesis of graphene has been detailed previously [37]. The carbon nanotubes used in this work were single wall nanotubes (SWNTs) procured from Unidym, Inc. (Sunnyvale, CA). They were grown using the High Pressure Carbon Monoxide Process (HiPCO) [38]. The syntheses of copper oxide nanowires and polypyrrole nanofibers are chemical processes, which have been detailed in previous work [34], [39].

The optimum DEP assembly parameters (dispersion concentration, applied voltage and frequency) were determined heuristically, and are comparable to values found in the literature. The parameters tabulated in Table 1 demonstrated the most reliable assemblies across the given 3 µm gap. For each material to be assembled, the appropriate signal was applied in parallel across the chosen set of electrodes. The dispersion was placed covering the chip with a syringe for 1 minute, after which the dispersion was removed from the chip with compressed air. This process was executed sequentially for each material assembled. After each assembly, the resistance was checked at each assembly site to ensure assembly occurred. While the materials in this paper are primarily semiconductors, the process is extensible to metallic and dielectric nanomaterials as well.

**Table 1:** Optimum DEP assembly parameters.

Material	Medium	Dispersion concentration	Assembly Voltage (pk-pk)	Assembly Frequency Range (Nominal value)
Carbon Nanotubes	DI Water	≈5 mg/ml	5 V	1 MHz–10 MHz (1 MHz)
Reduced GrapheneOxide	DMF	≈1 mg/ml	5 V	100 kHz–500 kHz (100 KHz)
Polypyrrole	Ethanol	≈1 mg/ml	7.5 V	100 kHz–500 kHz (100 KHz)
CopperOxide	DI Water	≈50 µg/ml	5 V	50 kHz–500 kHz (1 MHz)

For testing gas sensitivity of the heterogeneous array, a custom-made environmental chamber was constructed with electrical feedthroughs and gas ports. For saturated gas measurements, compressed dry air was bubbled through the liquid phase of the target analyte and fed to the chamber. For concentration measurements, the desired concentration of vapor (100–10 ppm) is introduced into the test chamber using an Environics 4040 computerized gas mixing system using N_2_ as the dilution gas. A Keithley 6140 source meter monitored the DC impedance of each assembly in the array. A custom-made printed circuit board served as the interface between the source meter and the assembled chip and handled the time-multiplexing of the impedance measurements.

## Results and Discussion

Resistive assemblies were achieved at all the electrodes (100% yield) where assembly was attempted for all the four types of nanomaterials. However, the yield of the working micro assemblies was lower at 65% due to process issues such as lithographic, packaging or soldering errors; issues that could be easily optimized in the future. For example, in some cases, where the lengths of nanomaterials were smaller than the electrode gap, we see an interconnected network of nanomaterials bridging the gap (see carbon nanotubes and PPy in Figure 3). Also, in the case of CuO nanowires, we see smaller CuO nanowires (refer to the figure) assembled only on one side of the electrode pair, however they do not contribute to chemiresistive responses since they do not bridge the electrodes. Sources of failed sensors include lack of wire bond integrity, lithographic errors, and corrosion of electrodes during assembly. In the case of rGO, poisoning of the DEP assemblies occurred when immersed in the dispersions for subsequent assemblies of PPy in ethanol rendering them less sensitive. No effect of cross-contamination or spurious assembly of unwanted nanomaterials if any, was observed or evidenced at nonspecific electrodes which is one of the key strengths of the proposed approach. The cross-contamination measurement was arrived at on the basis of impedance measurements between all the electrode pairs in between the sequential steps of assemblies of different nanomaterials. There was no change in the impedance (measured within the error of the instrument ∼10^−2^ ohms) that were not selected for DEP. This includes measurement of electrodes with prior assemblies on them. This feature can be attributed to two reasons: first, that electric field is applied locally to only those electrodes where assembly is desired, and second that the dielectrophoretic force acts only on nanomaterials in the dispersion and not on preassembled nanomaterials. Moreover, preassembled nanomaterials are strongly held by Van der Waals forces to their electrodes, and were not observed to disassemble during subsequent assemblies. One point should be made here which is that since no images or material characterization was done at each electrode sites in between assemblies, one cannot really claim that there is absolutely no cross-contamination. However from practical viewpoint of sensing application, the impedance measurement indicated no effect of any cross-contamination if there was one.

The resolution of assembly depends on the electrode spacing and can be made to reach nanometer dimensions. However an accurate characterization of the minimum resolution possible has yet to be done and could be the focus of future effort.

Our previous work with in situ monitoring of DEP assembled rGO via scanning probe microscopy has shown that the primary mechanism of sensing is due to redox reactions of the gas species modulating the charge carriers in the semiconductor assembly [40]. The same work also shows that the contact resistance is not a significant factor of the total sensor resistance. It is theorized that the same mechanism is responsible for the sensing action in the other assemblies however further investigations are necessary and is a focus of ongoing investigations.

**Figure 4:**
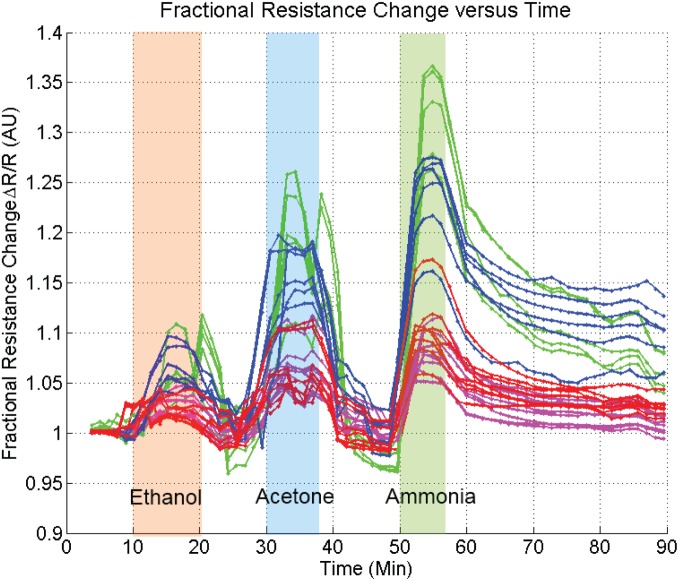
Fractional resistance changes to ethanol, acetone, and ammonia (20% w/w in water) in that order of introduction. The concentration is the vapor pressure of each analyte (6 kPa, 25 kPa, and 20 kPa). Green corresponds to RGO, blue to CNTs, red to copper oxide, and magenta to polypyrrole.

Sensor readout requires monitoring resistivity across each electrode where different nanomaterials were assembled. This was achieved through time-multiplexed measurements. A probe current of 1 µA was applied to each assembly, and the resulting voltage was measured. All forty assembly sites are measured every thirty seconds. The raw sensor response to common vapor environments is shown in Figure 4 and summarized in Table 2. The sensor was exposed to vapor pressures of three representative analytes: ethanol, acetone and ammonia. Response times for each type of chemiresistor were within minutes, and limited by the rate of gas introduction rather than the responsivity of the sensors. Recovery times are also on the order of minutes, except in the case of ammonia for which recovery times were on the order of an hour. The baseline impedances vary an order of magnitude and can range from 1–10 Kohms for CNT, 1–10 Kohms for RGO, 1–10 Mohms for CuO and 10–100 Mohms for PPy in response to different gases. The fractional resistance change is provides a consistent response to gases from 2% to 35% with rGO being the most sensitive. We have previously shown that the contact resistance is not a significant contributor to either the resistivity of the assembly or the chemiresistive response [40].

**Table 2:** Average response magnitudes of sensor element material to different analytes.

Analyte	CNT	rGO	Copper Oxide	Polypyrrole
Ethanol	7.5%	7.5%	2.5%	2.5%
Acetone	15%	20%	7.5%	5%
Ammonia	22.5%	32.5%	10%	7.5%

**Figure 5:**
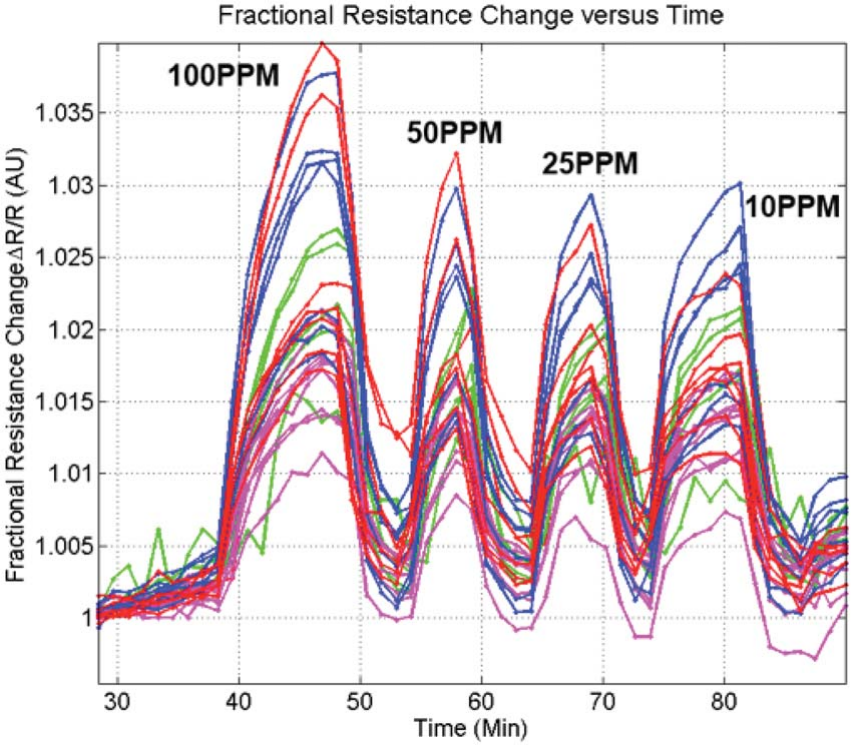
Concentration dependence of assembled sensors to ammonia vapor of varying concentrations.

Shown in Figure 5 is the concentration dependence of the sensor ensemble. After averaging and accounting for instrument noise, the detection limit for ammonia can be shown to be 400 ppb, 680 ppb, 880 ppb and 630 ppb for CNT, RGO, PPy and CuO DEP assemblies, respectively. However the standard deviation of individual responses at each sensing sites is quite large which must be improved in the future through further process optimization. These represent levels an order of magnitude better than the human detection limit. Higher sensitivities may be attained by integrating for a longer period at each element when cycling through assemblies. A trade-off exists between cycle time and sensitivity. A cycle time of thirty seconds was selected as it allowed for the adequate resolving of resistance profiles (like those shown in Figures 4 & 5), while also yielding acceptable detection limits.

The chemical sensing response to different vapor analytes demonstrates its ability to distinguish between gas species. The differences in the magnitude of the responses as well as their characteristic response time provides the necessary diversity and redundancy for robust detection of different vapors. Complex pattern recognition approaches can be employed for machine olfaction, when discrimination is desired among a larger range of targets in complex mixtures and backgrounds, which could be an area of future investigation.

While our prior work on sensing indicated that contact resistance was not a big factor in the mechanism of sensing [40], and the nanomaterials stayed in place mainly due to surface forces (e.g. van der Waals forces) with no observable change in baseline resistance with repeated use, there was no special effort made to make ohmic contacts between the nanomaterials and the underlying electrode. However, for other applications in electronics and sensing, one will be required to address the issue of reliable ohmic contact formations with each nanomaterial, which may become increasingly challenging as the diversity of nanomaterials integrated on the chip will increase. In the past, we had proposed a combination of electroless and electrochemical plating that selectively deposited metal (zinc, gold) around assembled nanomaterials, however the process was limited to carbon nanotubes [16], [35] and has yet to be explored for other nanomaterials reported in this paper. The approach is expected to work as long as the metals for ohmic contact formation can be electrodeposited and annealed. This will surely be an important matter to investigate for future research.

## Conclusion

This work demonstrates a throughput integration approach based on spatially controlled dielectrophoresis executed sequentially for each nanomaterial type to realize a scalable array of individually addressable assemblies of graphene, carbon nanotubes, metal oxide nanowires and conductive polymers on a single chip. The fabricated array was utilized in the detection of various gas species at PPB concentrations; thus illustrating the practicality and potential of such a platform. This integration platform can also be applied to optically, biologically or mechanically sensitive nanomaterials for the realization of, respectively, image sensors, bioassays, or tactile sensors. Future directions for the platform involve the expansion of the array dimensions to add more elements, the inclusion of a greater variety of sensor elements, and implementation of a pattern recognition engine to allow for the identification and quantification of gases. In terms of technology, issue of contact formation with diverse nanomaterials will also need to be addressed for other applications. Furthermore, since the process is naturally extensible to other substrates including top down fabricated CMOS-dies, it will enable hybrid CMOS-nano integration currently not possible with other approaches such as microcontact or nanocontact printing or nanoimprint lithography. This will form the basis of any future work.
